# Methylation and copy number profiling: emerging tools to differentiate osteoblastoma from malignant mimics?

**DOI:** 10.1038/s41379-022-01071-1

**Published:** 2022-03-28

**Authors:** Baptiste Ameline, Michaela Nathrath, Karolin H. Nord, Felix Haglund de Flon, Judith V. M. G. Bovée, Andreas H. Krieg, Sylvia Höller, Jürgen Hench, Daniel Baumhoer

**Affiliations:** 1 Bone Tumor Reference Center at the Institute for Medical Genetics and Pathology, University Hospital Basel, University of Basel, Basel, Switzerland; 2 Department of Pediatrics and Children’s Cancer Research Center, Klinikum rechts der Isar, Technical University of Munich, School of Medicine, Munich, Germany; 3 Pediatric Hematology and Oncology, Klinikum Kassel, Kassel, Germany; 4 Department of Laboratory Medicine, Division of Clinical Genetics, Lund University, Lund, Sweden; 5 Department of Oncology-Pathology, Karolinska Institutet, Solna, Stockholm, Sweden; 6 Clinical Pathology and Cancer Diagnostics, Karolinska University Hospital, Solna, Stockholm, Sweden; 7 Department of Pathology, Leiden University Medical Center, Leiden, The Netherlands; 8 Bone and Soft tissue Sarcoma Center, University of Basel, University Children’s Hospital Basel (UKBB), Basel, Switzerland; 9 Stadtspital Zürich, Institute of Clinical Pathology, University of Zürich, Zürich, Switzerland; 10 Institute for Medical Genetics and Pathology, Division of Neuropathology, University Hospital Basel, Basel, Switzerland

**Keywords:** Diagnostic markers, Bone cancer, Epigenomics

## Abstract

Rearrangements of the transcription factors FOS and FOSB have recently been identified as the genetic driver event underlying osteoid osteoma and osteoblastoma. Nuclear overexpression of FOS and FOSB have since then emerged as a reliable surrogate marker despite limitations in specificity and sensitivity. Indeed, osteosarcoma can infrequently show nuclear FOS expression and a small fraction of osteoblastomas seem to arise independent of FOS/FOSB rearrangements. Acid decalcification and tissue preservation are additional factors that can negatively influence immunohistochemical testing and make diagnostic decision-making challenging in individual cases. Particularly aggressive appearing osteoblastomas, also referred to as epithelioid osteoblastomas, and osteoblastoma-like osteosarcoma can be difficult to distinguish, underlining the need for additional markers to support the diagnosis. Methylation and copy number profiling, a technique well established for the classification of brain tumors, might fill this gap. Here, we set out to comprehensively characterize a series of 77 osteoblastomas by immunohistochemistry, fluorescence in-situ hybridization as well as copy number and methylation profiling and compared our findings to histologic mimics. Our results show that osteoblastomas are uniformly characterized by flat copy number profiles that can add certainty in reaching the correct diagnosis. The methylation cluster formed by osteoblastomas, however, so far lacks specificity and can be misleading in individual cases.

## Introduction

**Fig. 1:**
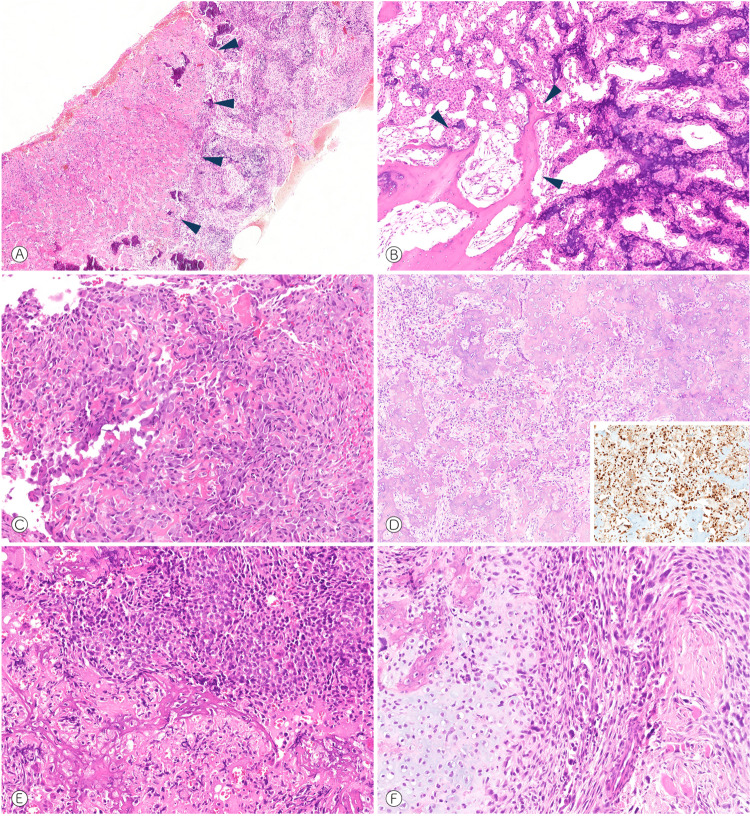
Distinctive histo-morphological features of osteoblastoma-like osteosarcoma. Histology of OB-like osteosarcoma show areas strongly resembling osteoblastoma (**A**–**C**) but merging with components with more obvious conventional osteosarcoma differentiation (**A**, arrowheads point to the OB-like differentiation) and destruction of pre-existing bone (**B**, arrowheads). Osteoblastomas present markedly similar morphology and usually show strong and consistent nuclear FOS expression (**D**). High-grade atypia of an osteoblastic (**E**) and chondroblastic (**F**) osteosarcoma are shown for comparison.

Osteoid osteoma and osteoblastoma are morphologically similar bone-forming tumors. Whereas osteoid osteoma is considered benign and generally does not exceed 2 cm in diameter, osteoblastoma behaves locally aggressive and can reach >10 cm in size. Osteoid osteoma is more common and represents 10–12% of all primary bone tumors, typically occurs in the long and small tubular bones and preferentially involves the cortex. Osteoblastoma is rare (<1% of all primary bone tumors) and mostly develops in the spine^1^. The most frequent bone-forming tumor is conventional osteosarcoma, an intraosseous high-grade sarcoma, that requires intense multimodal treatment and in patients with metastatic and/or recurrent disease is still associated with a poor outcome. Although the morphology usually differs significantly, the differential diagnosis between osteosarcoma and osteoblastoma can be challenging, especially in core needle biopsies. The rare osteoblastoma-like osteosarcoma variant is histologically defined by its similarity to osteoblastoma (Fig. 1). Due to the crucial differences in clinical outcome and treatment, reliable classification of bone-forming tumors is critical for providing adequate clinical care.

It has recently been shown that rearrangements of the transcription factor *FOS* and to a lesser extent also of its paralogue *FOSB*, represent highly recurrent driver mutations in osteoblastoma and osteoid osteoma^2^. These translocations result in gene fusions in which the partner gene is highly variable but contributes to the overexpression of the FOS/FOSB proteins^2,3^. As a consequence, immunostaining of FOS/FOSB has become a valuable surrogate marker and tool in routine diagnostics along with fluorescent in situ hybridisation (FISH). The gene fusion has been detected in the vast majority of osteoblastomas but around 5–11%^2,4^ lack evidence of these aberrations. Whereas immunohistochemistry is not specific for an underlying translocation and can be influenced by tissue preservation, a smaller fraction of osteoblastomas are characterized by homozygous *NF2* deletion and lack *FOS/FOSB* rearrangements^5,6^.

The identification of FOS/FOSB overexpression is helpful in routine diagnostics but as outlined is not present in all cases and can be identified also in a small subset of osteosarcomas. The availability of an alternative molecular marker would therefore be highly valuable. Recently, DNA methylation profiling has been shown to represent an alternative approach for the classification of brain tumors^7^. It correlates well with conventional tumor typing using histology and molecular genetics and has been shown to reproducibly identify new tumor subtypes that were indistinguishable before but differ in response to treatment and prognosis. Furthermore, methylation profiling using the Infinium Human Methylation450K BeadChip or Epic Array (850k) platform provides whole genome copy number changes which can point to specific gene amplifications/rearrangements, complex patterns of aberrations caused by chromoanagenesis and the degree of aneuploidy in general.

Methodologically, methylation profiling is based on the detection of 5-methylcytosines across CpG sites across the genome, of which the Infinium methylation arrays interrogate 450/850k per sample. The profile of an individual tumor is then compared with reference methylation classes that have been established using cases with unequivocal diagnoses (ground truth). This comparison is achieved using a supervised learning algorithm called random forest^8^. Such a classifier generates decision trees on randomly selected series of CpGs. Once the forest has been trained to recognize the reference samples, each tree provides an individual prediction for the tested sample and the majority vote is selected. As soon as methylation classes for all tumor subtypes are established, any given sample should in theory be classifiable based on its methylome profile. This approach has been shown to provide a highly specific and reproducible classification for brain tumors^7^ and initial studies yielded promising results also for soft tissue and bone tumors based on the so-called ‘Heidelberg Sarcoma Classifier’^8^, although a validation study revealed variable performance depending on tumor type^9^. Thus, potentially, the methylation classifier can be a valuable diagnostic tool, provided its results are interpreted within the appropriate clinical, morphological and immunohistochemical context.

In this study, we set out to perform FOS/FOSB immunohistochemistry, FOS fluorescence in-situ hybridization as well as methylation and copy number profiling of 77 osteoblastomas and to compare our results with fibrous dysplasia cases and a large set of osteosarcomas including conventional, parosteal, central low-grade, periosteal and osteoblastoma-like subtypes.

## Materials and methods

### Patient samples

The archives of the institutes of pathology at the University Hospital Basel (CH), the Karolinska University Hospital (SE), the Skåne University Hospital (SE) and the department of Pathology at the Leiden University Medical Center (NL) were searched for osteoblastomas and osteoid osteomas with sufficient amounts of well preserved tumor tissue for molecular studies. In total, 77 osteoblastomas and two osteoid osteomas were retrieved. All tumors with available histology were reviewed by an expert bone tumor pathologist (DB), samples with a tumor content below 40% were excluded from the study. Clinico-pathological data are provided in the Supplementary Table S1.

### Fluorescence in situ hybridization (FISH) for FOS

Among the set of 77 osteoblastomas, 24 cases (from the Leiden University Medical Center) had already been investigated by FISH^10^. Another 28 samples lacked sufficient amounts of well preserved tissue sections. FISH analysis was therefore performed in 25 osteoblastomas as described previously^10^. BAC probes flanking the distal and proximal regions of the *FOS* gene included RP11-173A8 pooled with RP11-316E14 and RP11-361H10 pooled with RP11-368K8.

### Immunohistochemistry (IHC) for FOS

IHC was performed in 45 osteoblastomas and two osteoid osteomas as described previously^11^. 24 cases (from the Leiden University Medical Center) had already been immunostained^4^ and another eight tumours (from the Lund University Hospital) lacked sufficient amounts of well preserved tissue sections. All immunoreactions used a rabbit polyclonal antibody directed against the N-terminal region of FOS (clone, F7799, Sigma, St. Louis, Missouri, USA).

### DNA methylation data sets

All the 77 osteoblastomas and two osteoid osteomas included in this study were subjected to DNA methylation analysis based on the Infinium Human Epic Array (850k) platform (Illumina). 24 formalin-fixed paraffin embedded (FFPE) and eight fresh frozen samples yielded interpretable results whereas 41 samples failed to pass the quality controls, mostly due to limited tissue preservation. The remaining six cases had already been evaluated by 850k arrays and used to generate the reference class osteoblastoma of the ‘Heidelberg Sarcoma Classifier’. Additionally, FFPE samples from two parosteal osteosarcomas, two fibrous dysplasia cases, four osteoblastoma-like osteosarcomas and ten central low-grade osteosarcomas were also investigated (Supplementary Table S1). All arrays were hybridized and scanned externally in a fully automated platform (Life and Brain, Bonn). The raw methylation data were then processed together with data of several external datasets already published^8,9,12^, in order to reach a minimum of seven cases per tumor type (a threshold empirically shown sufficient to avoid clustering artefacts and enable adequate prediction^8^). Taken together, the dataset of DNA methylation arrays was composed of 99 high-grade osteosarcomas, 48 osteoblastomas, 22 parosteal osteosarcomas, 20 fibrous dysplasia cases, 15 central low-grade osteosarcomas, seven periosteal osteosarcomas and seven osteoblastoma-like osteosarcomas. All methylation data have been deposited in the European Nucleotide Archive under the study accession number: EGAD00010002279.

### Methylation array processing

Raw intensity data files (IDATs) from either the Methylation 450 K BeadChips or the Methylation Epic (=850k) BeadChips were processed with the R-package minfi (https://bioconductor.org/packages/release/bioc/html/minfi.html). Epic arrays were converted to a virtual 450 K array for joint normalization and processing of data from both platforms. Probes associated with known SNPs, non-CpGs and sex chromosomes were not taken into account for the evaluation. Moreover, samples with a mean of the detection *p*-value above 0.02 were discarded. Among the different functions of normalisation available, the ‘preprocessIllimuna’ function was used before generating the dimension reduction visualization whereas the ‘preprocessQuantile’ was preferred before deriving copy number profiles.

### Copy number profile

Copy number variations were inferred from the Infinium Human Methylation 450 K BeadChip or Epic Array platform using the R-package conumee (https://www.bioconductor.orgpackages/release/bioc/html/conumee.html), after the pre-processing of data described above. In the absence of paired normal samples, a set of 20 control samples were used as reference (tissue with reactive changes *n* = 10 and blood *n* = 10 obtained from the study of Koelsche et al., 2020^8^). The settings for copy number variation inference were as follows: a minimum number of probes per bin equal to 25; minimum bin size equal to 100,000 bp. Any sample with a background noise superior to 0.9 was excluded (six osteoblastomas, two osteoblastoma-like osteosarcomas, one fibrous dysplasia case and one conventional osteosarcoma despite suitable methylome data). All these samples were colored in dark grey in Fig. 3. Copy number events were called against their background noise. Any copy number variation inferior to a third of the individual background noise was considered non-significant and was thus filtered out. Finally, all copy number profiles have been reviewed individually. A score representing the percentage of the genome involved in any kind of copy number variation was then calculated (sum of the length of each CNV divided by the sum of the length of the 22 autosomes, after exclusion of the centromeric and telomeric areas).

### Unsupervised clustering

Batch effects related to the different array types (450k/850k) as well as the different tissue conditions (FFPE and fresh frozen) were corrected using the R-package ChAMP in order to correct the beta-values. The set of probes was then restricted to the top 25’000 most differentially methylated (based on the standard deviation) determine on 15’500 reference datasets mostly derived from the cancer genome atlas (TCGA) and gene expression omnibus (GEO) as previously described^13^. The full list of CpG IDs used for this study has been deposited on the European Nucleotide Archive mentioned above. Uniform manifold approximation and projection (UMAP) was performed on the results of a principal component analysis (20 PCs) calculated via the singular value decomposition of the beta methylation matrix. The R-package used for generating the graph can be found at (https://github.com/jlmelville/uwot). The settings used to generate the non-linear regression model were: PCA = 20; neighbors = 15. Once the model was established, each sample was subsequently colored depending on the percentage of genome recombined (described above) according to a blue-red gradient. The samples for which copy number profiling failed were labelled ‘N/A’ and colored black.

## Results

Our study included samples from 77 patients with osteoblastomas. There were 51 men and 26 females (ratio 1.96:1), the average age was 22.2 years (range 2–57 years). We additionally included two osteoid osteomas (both males, 13 and 20 years). Further clinico-pathological information are provided in the Supplementary Table S1.

### Immunohistochemistry and FISH

*FOS* gene rearrangements were investigated by immunostaining of the FOS protein (*n* = 45 osteoblastomas, *n* = 2 osteoid osteomas) and FISH of the *FOS* gene (*n* = 25). Consistent FOS expression was identified in 85% (*n* = 40) of osteoblastomas / osteoid osteomas, whereas 15% were immunohistochemically negative (*n* = 6) or not evaluable (*n* = 1). FISH analysis showed rearranged hybridization signals in 6/6 evaluable cases, all of which also demonstrated immunostaining (19 cases failed or were not informative). Thus, by combining both approaches, 85% of the osteoblastomas analyzed showed evidence of *FOS* rearrangement.

### Methylome analysis

DNA methylation profiling yielded evaluable results in 32 tumors, including 30 osteoblastomas and two osteoid osteomas. Twenty eight cases were *FOS* rearranged whereas the four others samples were not investigated by in-situ analyses due to the lack of sufficient well-preserved material (see Supplementary Table S1). Raw methylation data were then submitted to the ‘DKFZ Sarcoma Classifier’ for evaluation. Diagnoses were concordant with the histological diagnosis in only 34% of cases (*n* = 11, including the two osteoid osteomas) using the default settings of the classifier (prediction score > 0.9). Eleven additional cases had a correct prediction but their score was below the threshold. Among the remaining cases (*n* = 10), the classifier yielded other diagnoses with a weak prediction score (not significant) or no prediction at all.

### Copy number analysis

**Fig. 2:**
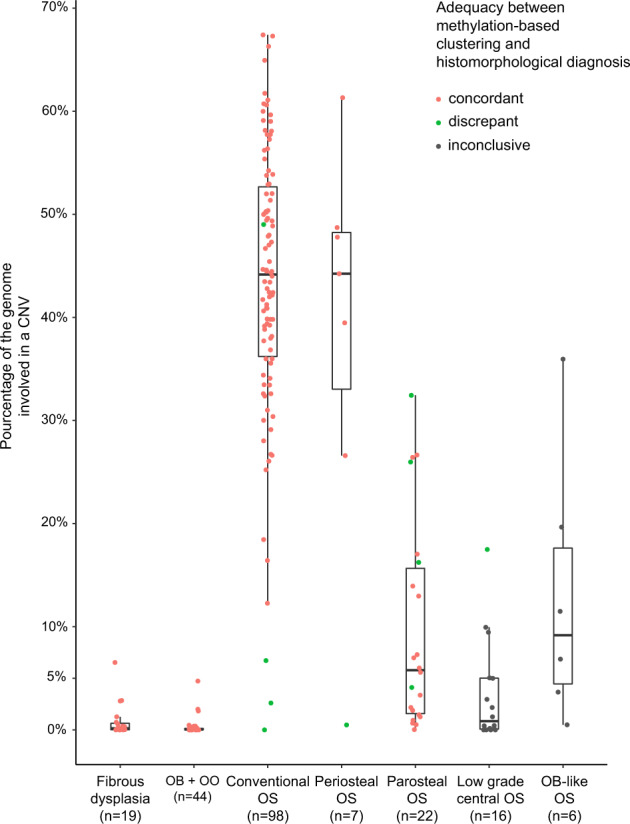
Distribution of the percentage of genomes recombined in different osteogenic tumor types. Following methylation-based clustering (UMAP), all specimens positioned outside their clusters are coloured green instead of red. In the absence of identifiable methylation clusters, low-grade central osteosarcomas and osteoblastoma-like osteosarcomas were colored black. Abbreviations: OB osteoblastoma, OO osteoid osteoma, OS osteosarcoma.

DNA methylation data were subsequently processed to obtain copy number profiles for each sample. In order to summarize the extent of copy number variations (CNV) into a single objective score, we counted the percentage of recombined base pairs over the 22 autosomes of each case. In addition to the osteoblastomas described, the analyses were complemented with data from recently published osteoblastomas as well as other bone-forming tumors (9, 12, 13). The dataset presented in Fig. 2 includes 42 osteoblastomas and two osteoid osteomas, 98 conventional osteosarcomas, 15 central low-grade osteosarcomas, 22 parosteal osteosarcomas, seven periosteal osteosarcomas, six osteoblastoma-like osteosarcomas and 20 fibrous dysplasia cases. On average, 0.28% (range: 0.00–4.71%; *n* = 42) of the genomes of osteoblastomas demonstrated copy number variations whereas the proportion of recombined genomes reached 43.42% (range: 0.00–67.41%; *n* = 98) in conventional osteosarcomas. Intermediate-grade periosteal osteosarcoma appeared to be as severely recombined as the high-grade osteosarcomas for all except one case of this small group (38.37%, range: 0.47–61.31%; *n* = 7). Low-grade central osteosarcoma and parosteal osteosarcoma, two subtypes known to harbor amplifications of the *MDM2* gene in 25–30% and >80% of cases^14^, respectively, displayed a limited amount of CNV: 3.62% (range: 0.03–17.50%; *n* = 15) for low-grade central osteosarcoma and 9.72% (range: 0.03–32.45%; *n* = 22) for parosteal osteosarcoma, respectively. Copy number variations in fibrous dysplasia cases affected around 0.84% of their autosome (range: 0.00–6.54%; *n* = 20). Finally, the series of osteoblastoma-like osteosarcoma showed an average score of 13.0% (range: 0.49–36.0%; *n* = 6) of their genome to be involved by copy number changes. However, the high amount of intertumoral variability indicates difficulties to clearly define this rare osteosarcoma subtype.

### UMAP-based classification

**Fig. 3:**
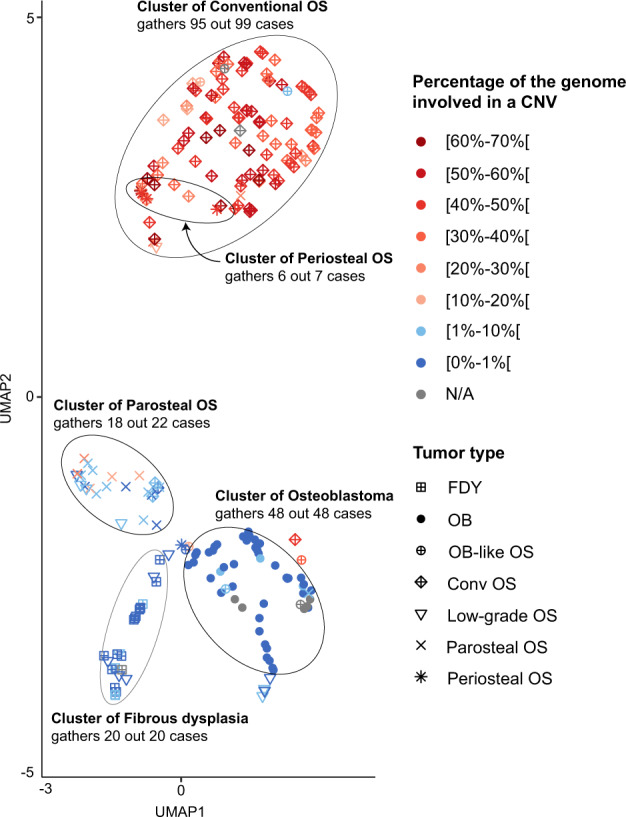
Methylation-based clustering of osteoblastomas and their mimics after uniform manifold approximation and projection (UMAP). Clustering of bone-forming tumors based on their methylation profile and colored according to the proportion of their genome affected by CNVs. Samples for which a quantifiable CN profile was available were colored black. Abbreviations: N/A not applicable.

**Fig. 4:**
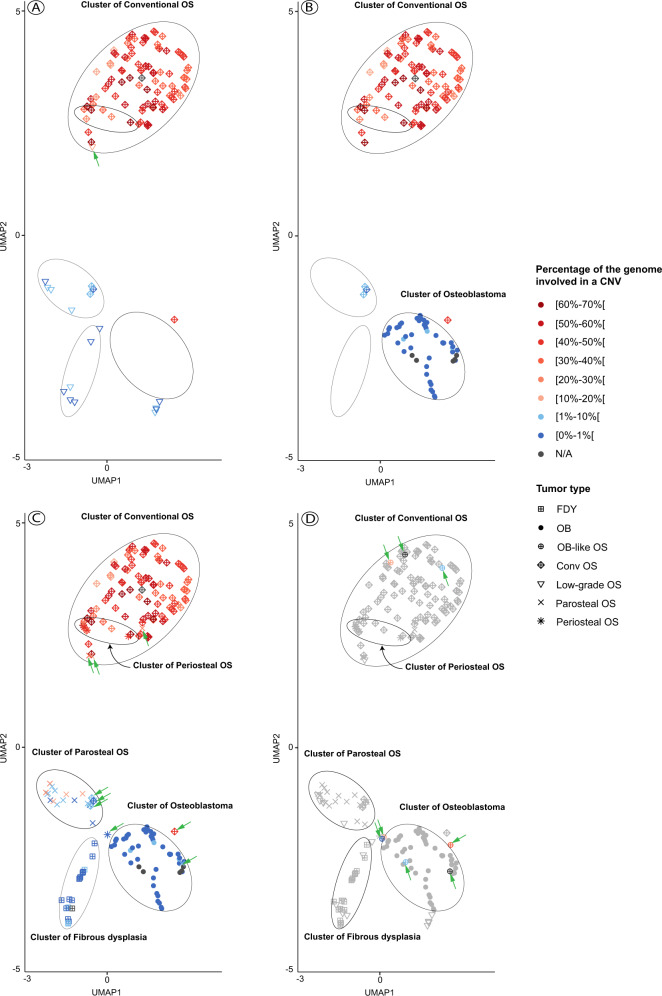
Selective comparison of different bone-forming tumors after nonlinear dimension reduction (UMAP). Each sample was colored according to the proportion of their genome affected by CNVs. The arrows depict the cases where both the amount of CNV and the methylation-based clustering diverge from the histopathological diagnosis. **A** Low-grade central osteosarcomas are compared to conventional osteosarcomas, (**B**) Osteoblastomas and osteoid osteomas are compared to conventional osteosarcomas, (**C**) All samples with an identifiable cluster are displayed. **D** In order to simplify the visualization, all samples except the osteoblastoma-like osteosarcomas are colored in grey. Osteoblastoma-like osteosarcoma are colored according to the proportion of the genome affected by CNVs.

Classification of bone tumors based on their methylation profile was also investigated using uniform manifold approximation and projection (UMAP) as an unsupervised clustering approach^15^. All tumors included in our study were put on a graph (Fig. 3) with the position depending on the similarity of the surrounding methylomes. The histomorphological diagnosis was not taken into account for generating the graph. Each case was then colored according to a blue-red gradient depending on the percentage of base pairs involved in copy number variations. At a first glance, five clusters were identified in which the vast majority of analyzed tumors aggregated. Benign/low-grade and intermediate/high-grade tumors were easily distinguishable (Fig. 3). However, the low-grade central and the osteoblastoma-like osteosarcomas were non-homogeneously distributed, indicating only limited similarities of their methylation profiles. As a second observation, the methylation and copy number profiles seemed to correlate well. Accordingly, most of the samples outside of their respective cluster were also the ones with an atypical copy number profile (Fig. 3). Indeed, the sole central low-grade osteosarcoma presenting a percentage of genome copy number changes >10% was also the only specimen localized among the intermediate/high-grade osteosarcomas (Fig. 4A). The same observation was made also for the sole outlier of periosteal osteosarcoma but with a reverse correlation. Regarding conventional osteosarcomas, four tumors were located outside their cluster of which three had a particularly flat copy number profile (Fig. 4A–C). Interestingly, the remaining fourth lesion, positioned at the periphery of the OB cluster, was also predicted to represent an osteoblastoma with the maximum confidence score given by the ‘DKFZ Sarcoma classifier’. However, its copy number profile presented an abundance of CNV (49%) hardly compatible with a benign tumor and no *FOS/FOSB* gene fusions were detected by RNA sequencing. Despite a typical histology of conventional osteosarcoma, this tumor seems to represent an OB-like osteosarcoma on a molecular level. Similarly, three parosteal osteosarcomas with some of the highest CNV scores in this class were positioned within the cluster of conventional osteosarcoma (Fig. 2 and Fig. 4C). The ‘DKFZ Sarcoma classifier’ does not yet include an established methylation class for this tumor type explaining why 13/15 tumors did not reach prediction scores >0.9. Two cases were predicted to represent conventional osteosarcomas (with the maximum confidence score) and both belonged to the three outliers described.

Taken together, although several tumor classes tended to form clusters, there was considerable overlap for individual tumor types. Indeed, periosteal osteosarcomas and conventional osteosarcomas could not be distinguished based on their methylation profile alone which was also true for low-grade central and parosteal osteosarcomas (Fig. 3). Moreover, the copy number profiles for each of these pairs did not appear useful in further stratification as both low-grade central and parosteal osteosarcomas can carry amplifications of *MDM2* whereas periosteal and conventional osteosarcomas generally display highly recombined profiles^1^. The presence or absence of *MDM2* amplification in the group of low-grade osteosarcomas was not sufficient alone to explain the fragmentation of this tumor class (Supplementary Fig. S1). However, we found most of the *MDM2*-amplified low-grade central osteosarcomas in close proximity to the parosteal osteosarcomas whereas none were positioned next to fibrous dysplasia cases (potential histologic mimics of low-grade osteosarcomas) and only one at the periphery of the osteoblastoma cluster.

A remarkable case initially diagnosed as an osteoblastoma showed a high similarity with the methylation class of giant cell tumors of bone (GCTB). Submission of the methylation data to the ‘DKFZ Sarcoma Classifier’ returned unclassified (confidence score below the threshold) although the class of GCTB was suggested as the closest match. Immunohistochemistry using the mutation-specific H3F3A (G34W) antibody indeed confirmed the diagnosis of GCTB and thus the methylome-based classification. The tumor histologically showed only few giant cells and abundant reactive new bone formation which resulted in a morphologic misinterpretation.

By reducing the scope of possibilities to the comparison of two or three individual methylation classes, important distinctions could be made: osteoblastomas and conventional high-grade osteosarcomas formed clearly separate clusters including only few mislocalized cases (Fig. 4B). In all except one tumor, the independent evaluation of the corresponding copy number profiles supported the methylation-based classification and underlined the rarity of mislocalized samples. 96% of high-grade osteosarcomas (95/99) and all osteoblastomas (48/48) could be distinguished when using both methylation and copy number profiling. However, when using copy number profiles only and applying an arbitrary cut-off score of 10% of the genome to be affected by copy number alterations, almost the same number of tumors were discernible (96/99) without taking into account additional methylome data. The three remaining high-grade osteosarcomas had exceptionally flat copy number profiles and all co-localized with parosteal osteosarcoma when evaluated by methylation-based clustering (Fig. 4B) despite unequivocal high-grade features on histology.

Notably, five FOS/FOSB-negative osteoblastomas coming from the study of Lyskjaer^9^ were also properly positioned within the osteoblastoma cluster. Secondly, low-grade central and high-grade osteosarcoma could be distinguished as well (Fig. 4A). Although the low-grade osteosarcomas were inconsistently distributed on the graph, these tumors remained easily discernible from conventional osteosarcomas. Unfortunately, even by reducing the number of tumor types evaluated, the described approach could not distinguish osteoblastoma-like osteosarcoma from osteoblastoma in 4/5 evaluable cases (Fig. 4D) but 5/6 cases lacked FOS expression and the remaining tumor showed only focal positivity. The copy number profiles in the high-grade tumors were all heavily rearranged, the two included low-grade osteoblastoma-like osteosarcoma showed a lower amount of copy number changes (6.85% and 11.5%, respectively).

## Discussion

The role of methylation-based non-brain tumor classification is an ongoing matter of debate^7,16^. One of the most promising and advanced applications in bone and soft tissue tumors is the ‘DKFZ Sarcoma classifier’. Despite adequate predictions in up to 83% of cases, critical reports have been published warning about the potentially misleading results of this approach, particularly if used by non-specialized pathologists^9^. With this study we suggest an alternative and additional use of DNA methylation profiling data.

Genome-wide copy number profiles can easily be derived from the Illumina Epic Array and used to challenge the prediction based on methylation profiles. In the current study, we used a score to assess the amount of copy number variations in individual genomes. Obviously, not all kinds of rearrangements can be identified by this approach, e.g. balanced translocations that leave the copy number profile unchanged. Nevertheless, this simple statistical approach proved valuable to complement the methylation-based tumor classification. Copy number profiling was highly effective in discriminating high and intermediate-grade malignant from low-grade malignant / locally aggressive / benign bone forming tumors. Additionally, one could imagine implementing an automatic detection of specific CNVs (such as *MDM2* amplifications in low-grade osteosarcoma) to further enhance diagnostic accuracy.

The current version of the ‘DKFZ Sarcoma Classifier’ (v12.2) aims to identify and distinguish 54 different bone and soft tissue tumor types by individual methylation classes^8^. Since the number of tumor samples used to generate the methylation classes varies and the current WHO classification differentiates >170 tumor subtypes, the performance of the classifier still has predictable limitations. The methylation class osteoblastoma has been generated by including methylome data of only seven tumors which might not be sufficient to capture the complete methylome spectrum of this benign and bone forming tumor (www.molecularneuropathology.org/mnp). Furthermore, it has been described that the classifier is less sensitive using FFPE samples compared to fresh frozen tissue^9^. All of these aspects might have contributed to the fact that only a third of cases in our study were correctly predicted to represent osteoblastomas (score ≥ 0.9) by the classifier. However, when taking into account also (or only) the copy number profiles, the diagnosis could be confirmed in 97% of cases. Methylation-based clustering also appears to be an interesting tool to visualize the similarity of methylation profiles between individual tumors using nonlinear dimensionality reduction (UMAP). In this work, we showed that osteoblastomas form a distinct cluster clearly separated from conventional osteosarcoma. Other osteogenic tumor types were also distinguishable such as low-grade central osteosarcoma from conventional osteosarcoma or osteoblastoma from periosteal osteosarcoma but these differential diagnoses are generally easy to render microscopically and in the clinico-radiological context and do not require additional genetic analyses. The identified clusters correlated well with the expected copy number profiles of the individual tumors. However, the UMAP plots clearly showed limitations in that some rather similar tumors could not be distinguished including parosteal osteosarcomas from central low-grade osteosarcomas and periosteal from conventional osteosarcomas. The osteoblastoma-like osteosarcomas included in our study were mis-classified in 4/5 evaluable cases.

Besides methylation and copy number profiling, immunostaining of FOS as well as the detection of a *FOS* rearrangement by FISH represent robust diagnostic tools as 85% of our osteoblastomas were shown to be positive in at least one of these in-situ analyses. Notably, five osteoblastoma-like osteosarcomas were immunohistochemically completely negative for FOS, the remaining case of which we included two manifestations was focally positive in one sample and negative in the other. All the FOS/FOSB-negative osteoblastomas (*n* = 5, all published previously by Lyskjaer et al.^9^) evaluated by methylome analysis in this study were properly positioned within the cluster of osteoblastomas. This suggests that the approach shown here can indeed help in discriminating osteoblastoma from conventional osteosarcoma even in the absence of a detectable *FOS*/*FOSB* fusion, representing a useful complementary tool. Although highly desirable, the molecular characterization of osteoblastomas relying on the expression of FOS/FOSB by IHC can be misleading in some cases. Indeed, expression of FOS has been documented in a small fraction of conventional osteosarcomas (<14%) although the immunoreactivity was usually focal^11^. Moreover, previous studies described osteoblastomas rich in epithelioid appearing cells that did not carry *FOS*/*FOSB* gene fusions^6,17^. In contrast to these observations, a small series of six epithelioid osteoblastomas of the jaws were described as all showing a strong nuclear FOS staining^18^. In our series, we identified only three tumors that showed a convincing epithelioid morphology whereas in other cases larger and epithelioid appearing cells were only a focal finding in otherwise conventional osteoblastomas. One of the three cases had insufficient amounts of well preserved DNA so the methylation array was not evaluable. Both other cases, however, were immunohistochemically strongly positive for FOS and co-localized with conventional osteoblastoma when evaluated by methylation-based clustering. The fractions of their genomes involved in copy number alterations were <2%. Epithelioid osteoblastoma therefore remains a rare and poorly defined subtype that, at least from the very limited data presented here, seems to show comparable methylome and copy number profiles like conventional osteoblastoma.

In conclusion, immunohistochemistry against FOS/FOSB continues to represent the quickest, most cost-effective and reasonable diagnostic add-on to confirm the diagnosis of osteoblastoma and to exclude (osteoblastoma-like) osteosarcoma. As all bone-forming tumors generally require decalcification which in many institutes still relies on acid-based methods, a significant amount of biopsies will not be applicable to additional molecular analyses as shown also by the high rate of drop-outs in our study. In case of morphologically unusual cases or inconsistent FOS/FOSB analysis, however, methylation and copy number profiling is an emerging tool to apply that can provide valuable additional information to accurately classify individual lesions. It is furthermore likely that methylome-based classification will become more accurate when the set of tumors defining individual methylation classes is significantly enlarged and the resolution of methylome profiling improves. The 850k methylation arrays interrogate only about 3% of the CpG sites present in the genome and for the methylation-based clustering only 10–25 k of the CpG sites are currently used and evaluated. Whole methylome sequencing and more advanced algorithms to evaluate this data might result in a significantly more precise prediction even of rare tumor subtypes. In the near future, methylation profiling using real-time nanopore sequencing could furthermore provide results within 2–3 h which might change the perspective of how surgical pathology is performed in general since tissue sections and molecular data would be available at the same time^19^.

## Supplementary information

Supplementary materials - Legends and supplementary Figure S1

Supplementary Table S1

## Data Availability

All raw intensity data files (IDATs) from the Methylation Epic (=850k) generated during this study have been deposited in the European Nucleotide Archive under the study accession number: EGAD00010002279.
