# Supplementary material for: Methylation and copy number profiling: emerging tools to differentiate osteoblastoma from malignant mimics?
Source: Mod Pathol. 2022 Mar 28;35(9):1204–11. doi: 10.1038/s41379-022-01071-1 (PMC9424109; doi:10.1038/s41379-022-01071-1)
Supplement: Supplementary file 1 — Supplementary materials - Legends and supplementary Figure S1 [file 41379_2022_1071_MOESM1_ESM.pdf]

SUPPLEMENTARY MATERIALS

Supplementary Figure S1 : Clustering of several bone-producing tumor types based on their methylation profile and colored according to the amplification of *MDM2*.

Supplementary Table S1: Clinical features and metadata of all samples investigated.

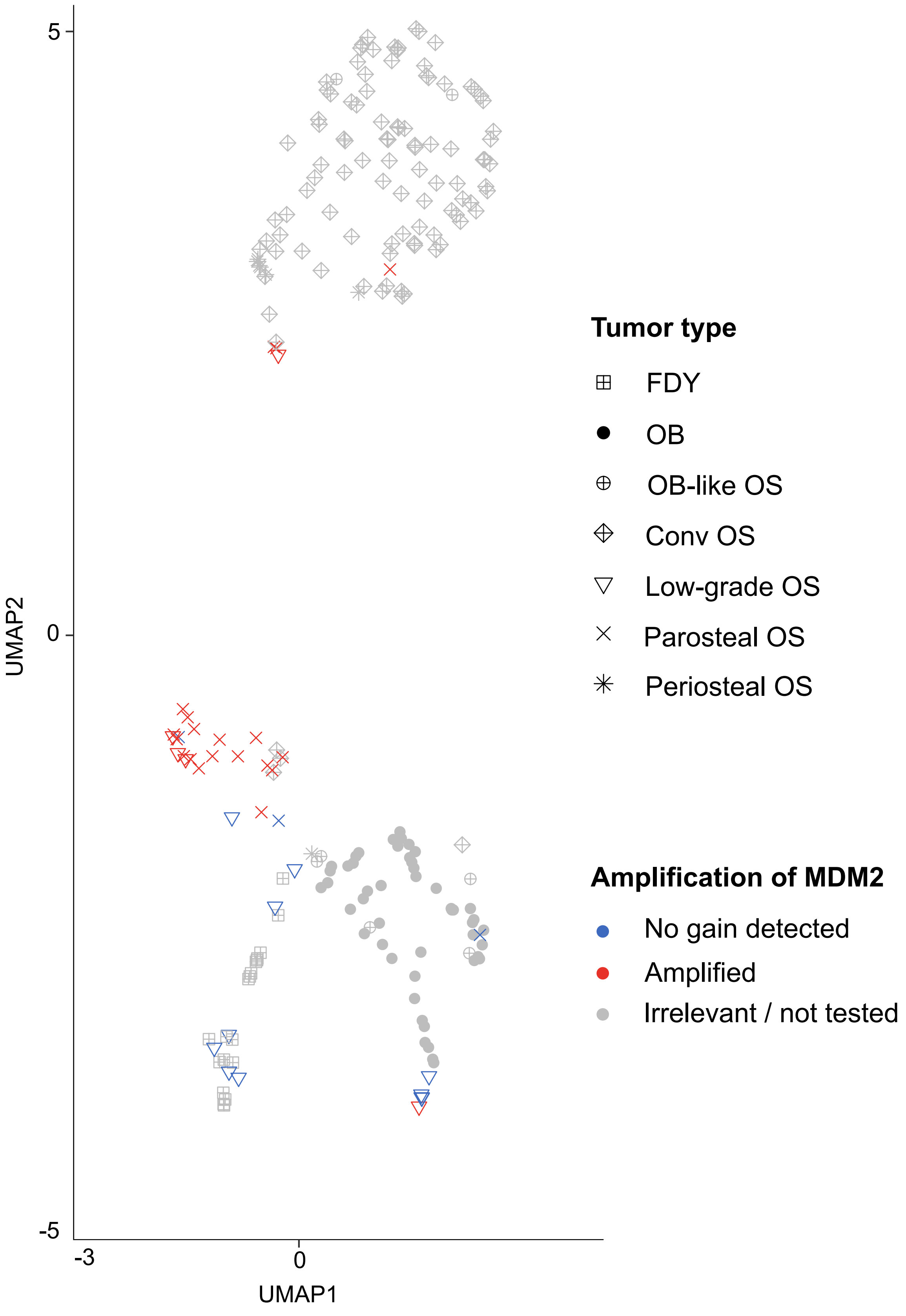

5
0
-5
-3
0
UMAP1
UMAP2
Tumor type
FDY
OB
OB-like OS
Conv OS
Low-grade OS
Parosteal OS
Periosteal OS
Amplification of MDM2
No gain detected
Amplified
Irrelevant / not tested
